# Causes of Visual Impairment Among the Registered Visually Disabled: A Retrospective Study

**DOI:** 10.7759/cureus.17988

**Published:** 2021-09-15

**Authors:** Nada Al-Yousuf, Haneen Alaali, Hassan M Alsetri, Hasan E Yusuf, Harish K Bhardwaj, Khatoon A Husain

**Affiliations:** 1 Ophthalmology, King Abdulla Medical City, Manama, BHR; 2 Ophthalmology, Salmaniya Medical Complex, Manama, BHR; 3 Chemistry and Biochemistry, University of California Los Angeles, Los Angeles, USA; 4 Ophthalmology, King Abdullah Medical City, Manama, BHR

**Keywords:** macular degeneration, congenital, hereditary, health policy, diabetes, glaucoma, diabetic retinopathy, blindness, low vision, visual disability

## Abstract

Purpose

To study the causes of visual impairment among Bahraini patients registered as visually disabled.

Materials and methods

A retrospective descriptive study of all patients referred to the Ministry of Social Development for visual disability from January 2014 to December 2019 was performed. Information recorded were age, gender, the cause of the visual impairment, and visual acuity in the better eye. If a patient had multiple ophthalmic diseases, the untreatable disease causing visual impairment was recorded. Patients were considered to have visual impairment according to World Health Organization criteria.

Results

A total of 484 Bahraini patients were included in the study. The mean age was 57.3 years of age ranging from 3 to 100 years; 63% of the total cases were males. The most common cause of visual impairment was diabetic retinopathy (DR) 201 (41.53%), followed by glaucoma 161 (33.26%). This is followed by hereditary and congenital disorders 34 (7.02%), glaucoma combined with DR 21 (4.34%), other retinal diseases 17 (3.51), retinitis pigmentosa 14 (2.89), optic atrophy 9 (1.86), corneal disorders 8 (1.65%), age-related macular degeneration 8 (1.65%), and others 11 (0.83%).

Conclusion

DR and glaucoma are the major causes of visual impairment among adults. Complications leading to visual impairment of both disorders are avoidable. Prevention measures to be taken control these diseases and prevent their morbidity. Congenital and hereditary disorders are the most common causes of visual impairment among children.

## Introduction

Visual impairment was ranked in 2015 as the third disability worldwide, after anemia and hearing loss [1]. Globally, the number of visually impaired of all ages is estimated to be 285 million, of which 39 million are blind. Interestingly, it was estimated that 80% of the total visual disability is due to preventable diseases [2]. Socioeconomic factors could explain 69.4% of the global variations in the prevalence of visual impairment [3].

In 2020, cataracts continued to be the leading cause of visual impairment of adults aged 50 years or more. It affects over 15 million adults, forming 45% of 33.6 million cases of global visual impairment. Refractive error is another large contributor to global visual impairment, constituting over 86 million individuals, forming 42% of 206 million cases of global visual impairment. Furthermore, diabetic retinopathy (DR), glaucoma, and age-related macular degeneration (ARMD) account for more than 19 million cases of visual impairment in 2020 [4].

In Eastern Mediterranean Region (EMR), uncorrected error of refraction and accommodation disorders, in addition to cataracts and glaucoma, constitute significant causes for visual impairment. Cataract, glaucoma, and macular degeneration were most prevalent in individuals aged 80 years and older in EMR both in 1990 and 2015. Refraction and accommodation disorders were more common among individuals aged 70-74 years in 1990 and 2015 [5,6].

Childhood visual impairment has a significant effect on the development of the child physically, mentally, and socially. The mortality rate is found to be higher in visually disabled children. Socioeconomic factors play a significant role in the prevalence of childhood visual impairment. Studies demonstrated that 1.4 million children with visual disabilities live in middle- and low-income countries [5,7].

This study is to demonstrate the causes of visual impairment in Bahrain among the different age groups and evaluate the possibility of preventing these disorders.

## Materials and methods

Bahraini citizens attending the eye clinic with low vision or blindness are referred to the Ministry of Social Development (MSD) in the Kingdom of Bahrain, for disability allowance. Patients with visual disabilities seen in the eye clinics of Salmaniya Medical Complex (SMC) and King Abdullah Medical City (KAMC) get referred to MSD as visually disabled. A retrospective descriptive study was carried out in December 2020 to identify the causes of visual impairment among all patients referred for visual disability. Patients’ case notes were reviewed from January 2014 to December 2019. If the cause of vision loss was treatable or can get rehabilitated medically, optically, or surgically, then the patient is not qualified as visually disabled. Patients with cataracts, keratoconus, correctable refractive error, recent vitreous hemorrhage, operable retinal disease, or recent diabetic maculopathy do not get referred to MSD as visually disabled. Approval was obtained from the Secondary Health Care Research Subcommittee (reference number 17/18) to review patients' records of SMC. Approval was also obtained from the Habib Medical Group Research Board (reference number RC19.02.27), to review patients’ records of KAMC. The study adhered to the Declaration of Helsinki guidelines. The nature of the study, being retrospective, did not require patients’ consent by both approval bodies. Measures were taken to ensure the patients’ anonymity and confidentiality.

The information recorded were demographic data such as age and gender, the cause of the visual impairment, and visual acuity in the better eye. For patients with multiple ophthalmic problems, the untreatable disease causing visual impairment was recorded. Patients were considered in our study to have visual impairment according to World Health Organization criteria. Visual impairment was divided into low vision and blindness. The low vision was further divided into mild visual impairment, which is visual acuity <0.5-0.3 (6/12 to 6/18). Moderate visual impairment, visual acuity 0.3-0.1 (6/18 to 6/60). Severe visual impairment, visual acuity 0.1-0.05 (6/60 to 3/60). Patients were considered to have blindness if the visual acuity was worse than 0.05 (3/60), or if they had visual field less than 10° around central fixation [7,8].

Patients do not get “labelled” as visually disabled unless they are examined by a consultant ophthalmologist with at least 10 years of experience. A thorough ophthalmology examination was conducted. The anterior segment of the eye was evaluated using a slit-lamp biomicroscope (Topcon, Japan). The posterior segment was examined using an indirect ophthalmoscope (Keeler, UK) and a +20 D fundus lens (Volk, Germany).

Recorded causes of visual impairment included untreatable DR, advanced glaucoma with optic disc damage, hereditary eye diseases, congenital abnormalities, inoperable corneal disorders, old trachoma, optic neuropathy, untreatable disorders of the retina and the choroid, trauma, toxicosis, and amblyopia. Congenital abnormalities referred to abnormalities in ocular structure and function caused by congenital reasons within one year after birth. Toxicosis referred to ocular changes caused by chemicals or drugs. Diseases with five or fewer patients were grouped together in one category named “others.” This category included trauma, toxicosis, trachoma, and amblyopia.

Data were entered and analyzed using Statistical Package for Social Sciences (SPSS) for Windows, version 21 (IBM Corp, Armonk, NY, USA). Descriptive statistics was performed using numbers and percentages to show nominal and categorical data.

## Results

A total of 484 Bahraini patients were included in the study. Patients with missing diagnoses were excluded. The mean age was 57.3 years ranging from 3 to 100 years. There was male preponderance forming 63% of the total cases. Table 1 shows the basic demographic characteristics in terms of age groups and gender.

**Table 1:** Demographic characteristics (age groups and gender)

Age	Total no. ( %)
0–16	20 (4.1)
17–29	25 (5.1)
30–39	64 (13.2)
40–49	49 (10.1)
50–59	76 (15.7)
60 and above	250 (51.6)
Gender	Total no. (%)
Males	306 (63.2)
Females	178 (36.7)

A total of 25 patients (5.17%) had visual impairment attributed to visual field restriction. There were 36 (7.44%) patients with mild visual impairment. One hundred and fifty-two (31%) patients had moderate visual impairment, 19 (3.9%) patients had severe visual impairment, and 252 (52%) patients were legally blind (Table 2).

**Table 2:** Patients with different categories of visual impairment VF: visual field; BCVA: best-corrected visual acuity.

Visual impairment category	BCVA in the better eye	No.	Percentage
VF < 10°	≥0.5 ( 6/12)	25	5.1
Mild	<0.5-0.3 (6/12-6/18 )	36	7.4
Moderate	<0.3-0.1 ( 6/18–6/60)	152	31.4
Severe	<0.1-0.05 ( 6/60–3/60)	19	3.9
Blindness	<0.05 (3/60)	252	52

**Figure 1:**
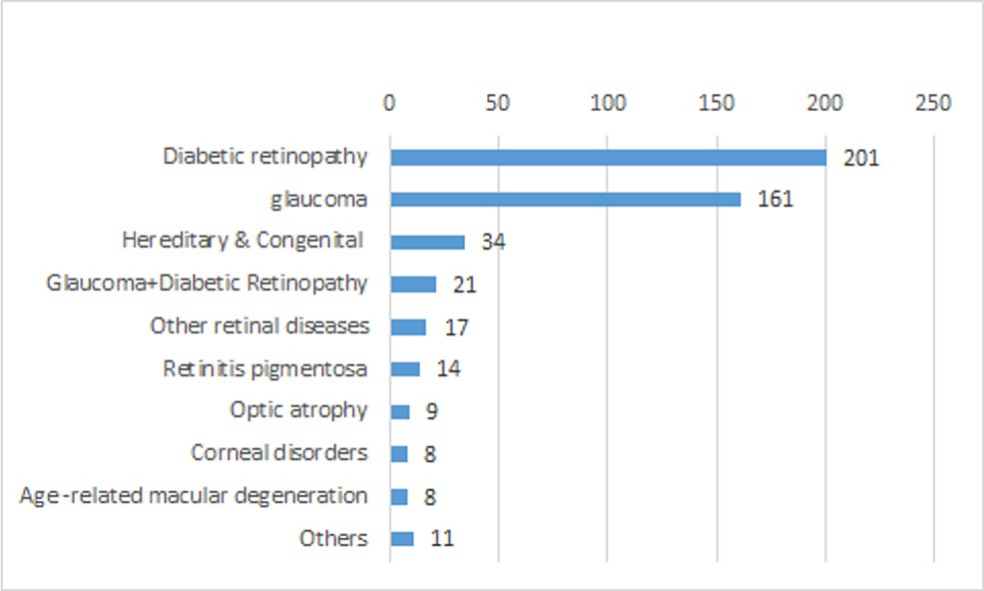
Causes of visual impairment among the registered visually disabled in the Kingdom of Bahrain.

Figure 1 shows the causes of visual impairment. The most prevalent cause of visual impairment was DR, 201 (41.53%), followed by glaucoma, 161 (33.26%). Other causes were hereditary and congenital disorders, 34 (7.02%), glaucoma combined with DR, 21 (4.34%), other retinal diseases, 17 (3.51), retinitis pigmentosa, 14 (2.89), optic atrophy, 9 (1.86), corneal disorders, 8 (1.65%), ARMD, 8 (1.65%), and others, 11 (0.83%).

**Figure 2:**
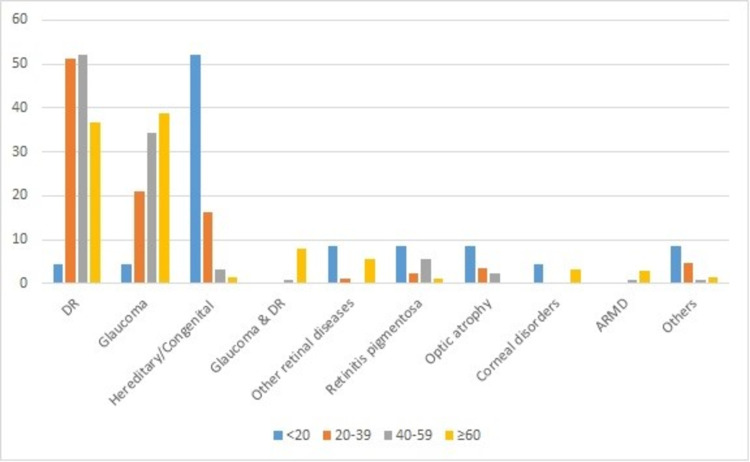
Causes of visual impairment in different age groups among patients registered as visually disabled in the Kingdom of Bahrain. DR: diabetic retinopathy; ARMD: age-related macular degeneration.

Figure 2 presents the causes of visual impairment in different age groups. In individuals aged <20 years, the leading cause of visual impairment was hereditary or congenital abnormality (52.1%). DR was the leading cause of visual impairment in the age groups 20-39 and 40-59, forming 51.16% and 52%, respectively. DR forms 4.35% of visual impairment in individuals under 20 years. The most common cause of visual impairment for adults aged ≥60 years was glaucoma (38%) followed by DR (36.8%).

## Discussion

Causes of visual impairment differ geographically, depending on the prevalence of ocular disorders in different parts of the world. Moreover, variations exist in the same country in different time periods. This is ascribed to the distribution of ocular disorders and systemic diseases causing visual impairment. The existence of such diseases is attributable to healthcare availability, advances in medical and surgical management of ocular disorders, public awareness, and socioeconomic status.

A previous study was done in the Kingdom of Bahrain collecting data from out-patient records. It was found that cataract used to be the most common cause for visual impairment forming 52%. DR, however, formed only 5% of the total cases [9]. DR is taking the lead in the current study as the major cause of visual impairment. This is likely to be due to the rise of diabetes with the change in lifestyle in the Kingdom of Bahrain. Indeed, the Middle East has the second highest rate of increase in diabetes among all countries [10,11]. The incidence of diabetes in the Kingdom of Bahrain is 14.7% of the adult population [12]. This is considered significantly high compared to Europe (8.5% ) and North America (10.5%) [13,14].

A study was conducted in the Kingdom of Saudi Arabia (KSA) examining the trends of causes of visual disability. They found that cataract used to be the leading cause of visual impairment, then DR took the lead in Jizan and Taif, west of KSA [15]. This is consistent with the change in causes of visual impairment in Bahrain [9]. Moreover, another study from Al Baha, KSA reported that diabetes was the most common cause of visual impairment followed by glaucoma [16]. This is consistent with our finding. Despite the socioeconomic dynamics that took place in the region, cataract is still a major cause of visual impairment in different parts in the Middle East. Cataract is the most common cause of visual impairment in Qatar [17] and in parts of KSA according to studies done in Bisha, Arar, and Al-Jouf [18-20]. Iran reported cataract to be the most common cause of visual impairment followed by errors of refraction [21].

Because of glaucoma being a major cause of visual impairment in Bahrain over the past 3 decades [9], light is shed on the significance of this disease and the need for preventive measures. Genetics and aging population can be the accounting factors [22,23]. Glaucoma ranked second after cataract in Taif, KSA in individuals above 50 years [16]. Glaucoma was also found to be the second most common cause for visual impairment in Qatar [17].

Our study shows male predominance (63%). A similar study conducted in Kuwait studying the causes of visual impairment among registered Kuwaiti citizens showed the same finding [24]. In their study , there was predominance of males, forming (66%) of the total registered visually disabled. On the other hand, population-based studies from the region on visual impairment showed female preponderance [17,19,21,25]. One possible explanation is that the current study and the study done in Kuwait were both examining individuals who were officially registered as visually disabled. This entitled them to receive disability allowances from their governmental institutions. For this purpose, males were probably more keen to get registered as disabled in order to receive allowances to support their families. It would be interesting to see if this gender pattern changes if population-based studies are conducted.

ARMD was considered a major cause of visual impairment in Europe and North America [26,27]. In England, Evans and co-workers found that ARMD was the leading cause among those registered as blind or partially sighted [28]. Wong et al. in their systematic review and meta-analysis study confirmed that ARMD was more prevalent among Europeans compared with Asians and Africans [29]. Al-Ghamdi, in his review, found that in KSA, ARMD accounted for 0.6-8% of the total causes of visual impairment, in Oman it was 2.9%, whereas in Europe it was at least 19.5% [15]. In our study, ARMD was one of the least common causes of visual impairment, forming only 1.65% of all cases. Our finding goes with regional and global findings. Genetic, racial, and environmental factors are responsible for this variability [26,27,29].

In pediatrics and young adults below the age of 20, we found that hereditary and congenital causes are the leading causes of visual impairment. In Saudi Arabia, hereditary retinal disorders formed the main cause of visual impairment among children and young adults [30]. This is consistent with our finding. This could be explained by consanguineous marriages.

This study highlighted important health and social factors accounting for visual impairment in the Kingdom of Bahrain. DR and glaucoma among adults, and hereditary diseases among children were the main causes of visual impairment. The limitation of this study is that some patients had missing diagnoses; therefore, they got excluded. This is not uncommon in retrospective studies. The causes of visual impairment in this study may not represent the population of Bahrain. This is because those were patients who attended eye clinics, and found to be visually impaired. Indeed, some individuals attended the eye clinics for the purpose of getting registered as visually disabled.

## Conclusions

DR and glaucoma are the leading causes of visual impairment among adults in the Kingdom of Bahrain. This finding is significant because both disorders are preventable. We recommend more measures to be taken to screen diabetic patients for DR. This will ensure early detection and prevention of sight-threatening complications. In addition, steps should be followed to modify risk factors attributing to diabetes. Glaucoma has been a major cause of visual impairment in the Kingdom of Bahrain over the past few decades. Serious measures should be taken to screen adults for early detection and treatment of glaucoma. In children, the leading cause of visual impairment was congenital and hereditary. Campaigns are suggested to increase the public awareness of the effects of consanguinity. This study opens doors for future studies, such as population-based screening programs, in order to reveal unrecognized and unregistered visual disabilities.
